# T Cell‐Specific Deficiency of Src Homology 2‐Containing Protein Tyrosine Phosphatase 2 Ameliorates Psoriasis and Colitis by Promoting Treg Differentiation

**DOI:** 10.1002/mco2.70310

**Published:** 2025-08-01

**Authors:** Shuqiong Zhang, Zijun Ouyang, Zhidan Fan, Haiyan Sun, Haiguo Yu, Xingxin Wu, Yang Sun, Fenli Shao

**Affiliations:** ^1^ State Key Laboratory of Pharmaceutical Biotechnology Chemistry and Biomedicine Innovation Center (ChemBIC) School of Life Sciences Nanjing University Nanjing China; ^2^ Jiangsu Key Laboratory of New Drug Research and Clinical Pharmacy Xuzhou Medical University Xuzhou China; ^3^ School of Food and Drug Shenzhen Polytechnic University Shenzhen China; ^4^ Department of Rheumatology and Immunology Children's Hospital of Nanjing Medical University Nanjing China; ^5^ State Key Laboratory of Technologies for Chinese Medicine Pharmaceutical Process Control and Intelligent Manufacture Nanjing University of Chinese Medicine Nanjing China

**Keywords:** adoptive transfer colitis, autoimmune disease, psoriasis, Src homology 2‐containing protein tyrosine phosphatase 2, Treg

## Abstract

Psoriasis and ulcerative colitis are both autoimmune diseases with complex pathogenesis characterized by immune disorders. Src homology 2‐containing protein tyrosine phosphatase 2 (SHP2) is a non‐receptor protein tyrosine phosphatase that acts as a key regulator of immune cell‐mediated inflammation. Although studies have described the role of SHP2 in autoimmune diseases, its influence on the development of regulatory T cells (Tregs) was undefined, which plays a critical role in immune homeostasis. Here, we found that imiquimod (IMQ)‐induced psoriasis symptoms were milder in *Lck*‐Cre;SHP2^f/f^ mice than those in SHP2^f/f^ mice, including reduced inflammatory cell infiltration and keratinocyte proliferation. The reduced Th17/Treg ratio in psoriasis models in *Lck*‐Cre;SHP2^f/f^ mice suggests that SHP2 regulates the balance of Th17/Treg. In vitro, the deficiency of SHP2 promotes the differentiation of T cells into Tregs. In the model of adoptive transfer colitis, the SHP2‐deficient CD4^+^CD25^−^CD45RB^high^ T cells differentiated into a greater number of Tregs within the recipient mice, resulting in attenuated symptoms of colitis. Moreover, cotransfer experiments confirmed that the deficiency of SHP2 does not affect the immunosuppressive function of Tregs. These findings establish that SHP2 reduces Treg differentiation and further confirm that SHP2 inhibitors could be utilized in the treatment of autoimmune diseases.

## Graphical abstract

**•** SHP2 deficiency in T cells could alleviate both psoriasis‐like skin lesion and adoptive transfer colitis.

**•** SHP2 deficiency in T cells promotes the differentiation toward Treg, thus regulating the balance of Th17/Treg.

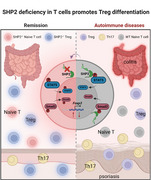

## 1 Introduction

Psoriasis is a prevalent chronic inflammatory skin disorder affecting approximately 2%–3% of the global population, characterized by dry, itchy, and raised cutaneous lesions with silvery scales, typically localized to the elbows, knees, scalp, and lower back [1, 2, 3]. Ulcerative colitis is an inflammatory bowel disease manifested by rectal bleeding and diarrhea that leads to structural and functional changes of the colorectal, resulting in impaired quality of life and disability [4, 5, 6]. Both psoriasis and colitis are immune‐mediated inflammatory conditions occurring in barrier organs and exhibit significant comorbidity, likely attributable to shared pathogenic mechanisms, including genetic susceptibility, environmental triggers, and dysregulated immune activation [4, 7, 8]. Similarly, these diseases involve coordinated interactions between adaptive and innate immune cells, cytokines, and stromal cells such as keratinocytes in psoriasis and epithelial cells in colitis, which collectively drive tissue damage and disease progression [9, 10, 11].

Tregs constitute a specialized subpopulation of helper T cells characterized by high expression of the IL‐2 receptor alpha chain CD25, which is indispensable for autoimmune regulation [12]. Additionally, the transcription factor Forkhead box protein P3 (Foxp3) governs immune response regulation and immunological tolerance maintenance, contributing to both Treg development and their subsequent suppressive function [13, 14]. Tregs suppress the activity of other effector immune cells mainly through direct interaction, cytotoxicity, and release of inhibitory cytokines such as IL‐10, TGF‐β, and IL‐35 [15, 16]. Depletion of specific regulatory CD4^+^ T cell subsets could induce the spontaneous onset of various autoimmune diseases [17]. Notably, distinct regulatory CD4^+^ T cell subsets exhibit organ‐specific tropism and selectively control different autoimmune conditions, while CD4^+^CD25^+^ Tregs are pivotal in maintaining tolerance and preventing autoimmune disorders [17]. Conceivably, several autoimmune diseases have been linked to reduced Treg function or quantity [18, 19].

Psoriasis is driven primarily by inflammatory signals mediated by Th1, Th2, Th17, and Th22 cells, which are regulated by Tregs in healthy individuals [20, 21]. In psoriasis, Treg dysfunction causes alterations in the Th17/Treg balance [22, 23]. Patients with psoriasis exhibit elevated frequencies of Th17 cells and Tregs in peripheral blood and lesional skin, whereas the Th17/Treg ratio correlates directly with disease severity [24, 25]. The immunological response of effector T cells and the pathogenesis of psoriasis are closely linked to Treg differentiation and functional integrity [26, 27]. Similarly, Tregs also play a powerful anti‐inflammatory role in colitis. As reported, Tregs are decreased in the peripheral blood of individuals with active inflammatory bowel disease [28, 29, 30]. Furthermore, Tregs help maintain intestinal mucosal homeostasis by inhibiting aberrant immune responses to gut microbiota or dietary antigens. In particular, Tregs produce IL‐10 and TGF‐β and prevent T cells from escaping tolerance mechanisms [31]. Collectively, these findings underscore the essential role of Tregs in preserving immune homeostasis in autoimmune diseases such as psoriasis and ulcerative colitis.

The protein tyrosine phosphatase non‐receptor type 11 (*PTPN11*) gene encodes SHP2, a non‐receptor protein tyrosine phosphatase (PTP) that is extensively expressed in cells across multiple organs and tissues [32]. SHP2 consists of two Src homology 2 (SH2) domains in the N‐terminal tail, a PTP catalytic domain, two tyrosine phosphorylation sites in the C‐terminal tail, and a proline‐rich region [33]. Upon extracellular signal activation, SHP2 transitions from its autoinhibited conformation to an active state through binding of phosphorylated substrates to its SH2 domains or via autophosphorylation at tyrosine residues [34]. Through direct modulation of immune response or inflammatory processes, SHP2 may function as a critical regulator of immune cell‐mediated inflammation [35] and has been implicated in gastrointestinal inflammation, neuroinflammation, liver inflammation, lung inflammation, as well as many autoimmune diseases including psoriasis, diabetes, ankylosing spondylitis, and so forth [34, 36, 37, 38, 39]. However, whether SHP2 affects Treg development remains unknown.

In this study, we observed that imiquimod (IMQ)‐induced psoriasis symptoms were alleviated in mice with T cell‐specific deletion of SHP2 compared to wild‐type mice. Flow cytometric analysis revealed a higher proportion of Tregs in these SHP2‐deficient mice. To determine whether SHP2 directly affects Treg differentiation, we conducted systematic in vitro and in vivo studies. In vitro, SHP2 deficiency promotes Treg differentiation. In an adoptive transfer model of colitis, SHP2‐deficient CD4^+^CD25^−^CD45RB^high^ T cells exhibited reduced pathogenicity and increased Treg generation compared to wild‐type cells. These findings confirm that SHP2 deficiency enhances T cell differentiation into Tregs under both in vitro and in vivo conditions. In a cotransfer model, SHP2‐deficient Tregs exhibited comparable immunosuppressive capacity to wild‐type Tregs, indicating that SHP2 regulates Treg differentiation without impairing their immunosuppressive function. Mechanistic studies revealed that SHP2 inhibits Treg differentiation by suppressing STAT5 phosphorylation downstream of IL‐2 signaling. Collectively, our research establishes that SHP2 directly inhibits Treg differentiation. Targeting SHP2 to modulate Treg differentiation could thus serve as a novel therapeutic strategy for autoimmune diseases such as psoriasis and colitis.

## 2 Results

### 2.1 SHP2 Deficiency in T Cell Alleviates IMQ‐Induced Psoriasis‐Like Skin Lesions in Mice

**FIGURE 1:**
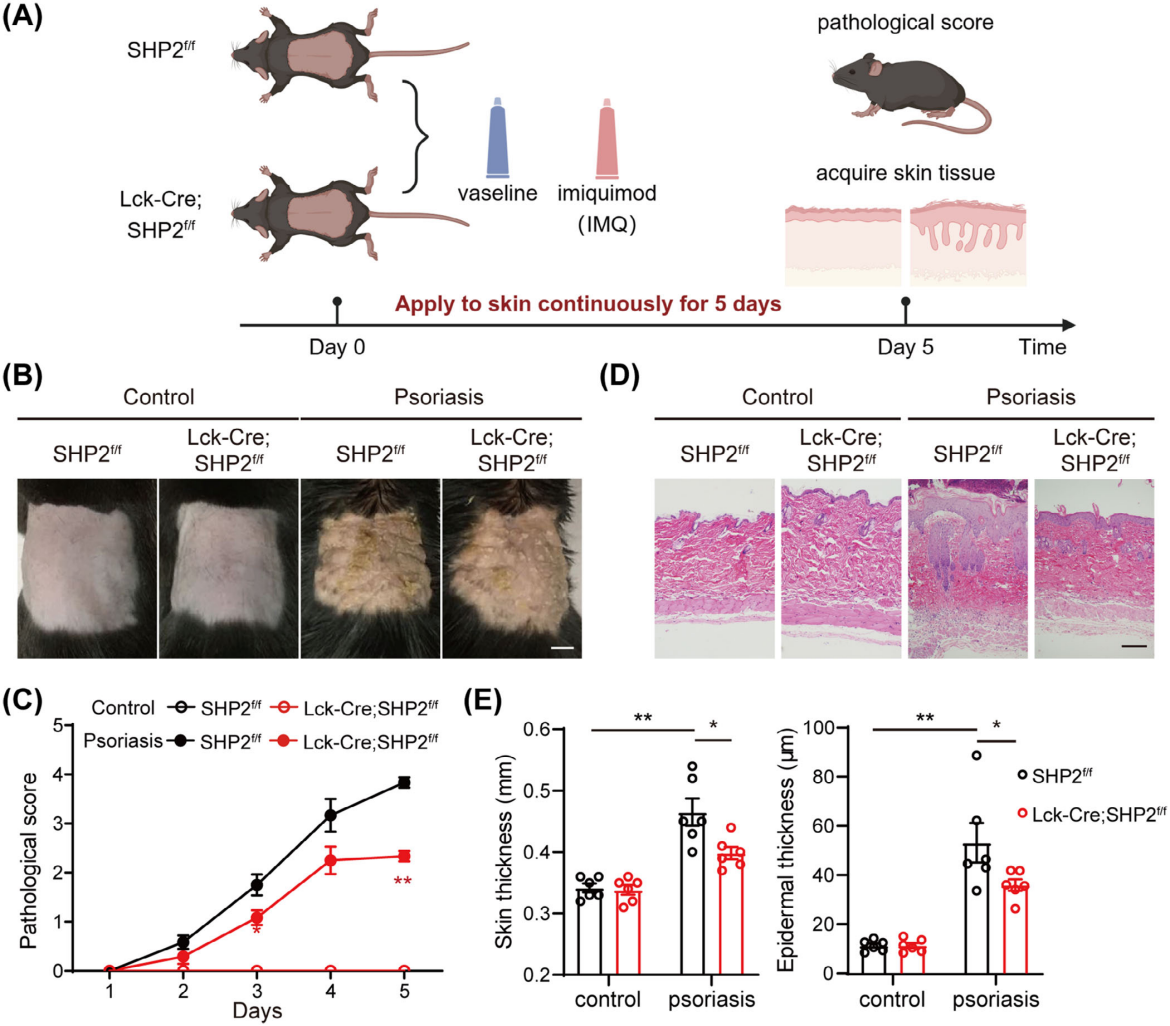
SHP2 deficiency in T cells alleviated psoriasis‐like symptoms in mice. (A) Modeling flow chart of IMQ‐induced psoriasis animal model. (B) Images of molding skin tissue in all groups, scale bar = 0.5 cm. (C) Pathological scores of mice in all groups. (D) HE staining of molding skin tissue in all groups, scale bar = 100 µm. (E) Quantitative statistical chart of skin and epidermal thickness in all groups. *n* = 6. *p < 0.05, **p < 0.01.

We induced psoriasis models through IMQ in SHP2^f/f^ and *Lck*‐Cre;SHP2^f/f^ mice and monitored the disease process for 5 days (Figure 1A). SHP2^f/f^ mice developed severe psoriasis‐like skin lesions characterized by erythema and scales, whereas *Lck*‐Cre;SHP2^f/f^ mice exhibited alleviated symptoms, including significantly reduced scaling coverage (Figure 1B) and lower pathological scores (Figure 1C). HE staining of skin tissues exhibited decreased thickening of the epidermis in *Lck*‐Cre;SHP2^f/f^ psoriasis mice compared to SHP2^f/f^ mice (Figure 1D,E).

**FIGURE 2:**
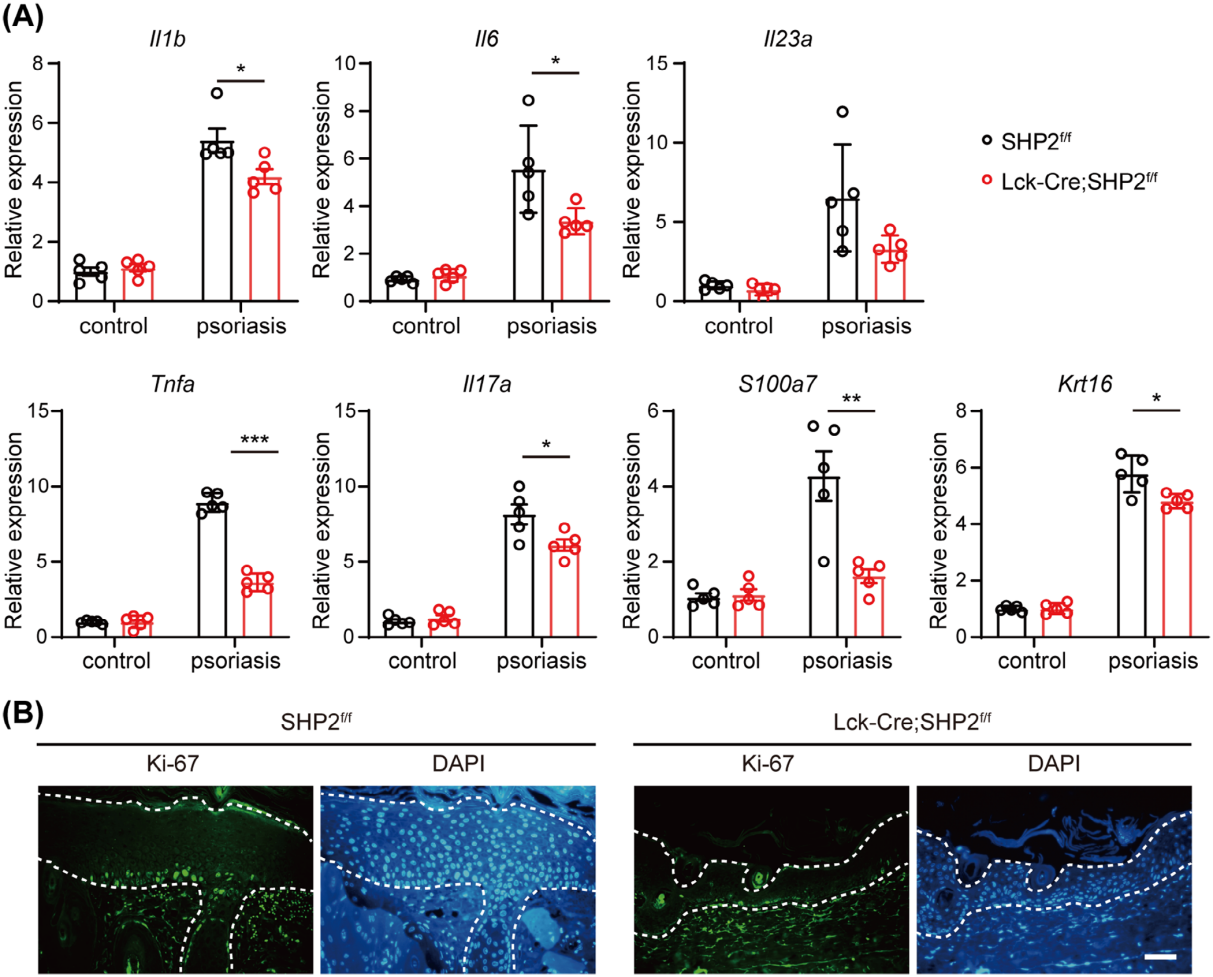
SHP2 deficiency in T cells relieved inflammation and keratinocyte proliferation in psoriasis‐like skin. (A) Gene expression of inflammatory factors and keratinocyte in molding skin tissue quantified by RT‐qPCR. *n* = 5. (B) Immunofluorescence results of Ki‐67‐positive keratinocytes in molding skin tissue, scale bar = 100 µm. *p < 0.05, **p < 0.01, ***p < 0.001.

Subsequently, we investigated the inflammation and keratinocyte proliferation condition. SHP2‐specific deficiency in T cells markedly reduced expression of Th17‐related immune genes such as *Il1b*, *Il6*, *Il23a*, and *Il17a*, as well as keratinocyte‐related genes *S100a7* and *Krt16* (Figure 2A), indicating relieved inflammation and abnormal keratinocyte proliferation. Immunofluorescence staining further revealed a higher frequency of Ki‐67‐positive keratinocytes in the epidermis of SHP2^f/f^ mice induced by IMQ than that of *Lck*‐Cre;SHP2^f/f^ mice (Figure 2B). Collectively, these results suggest that SHP2 deficiency in T cells alleviates psoriasis‐like skin lesions.

### 2.2 SHP2 Deficiency in T Cells Regulates the Immune Profile of Psoriasis‐Like Skin

**FIGURE 3:**
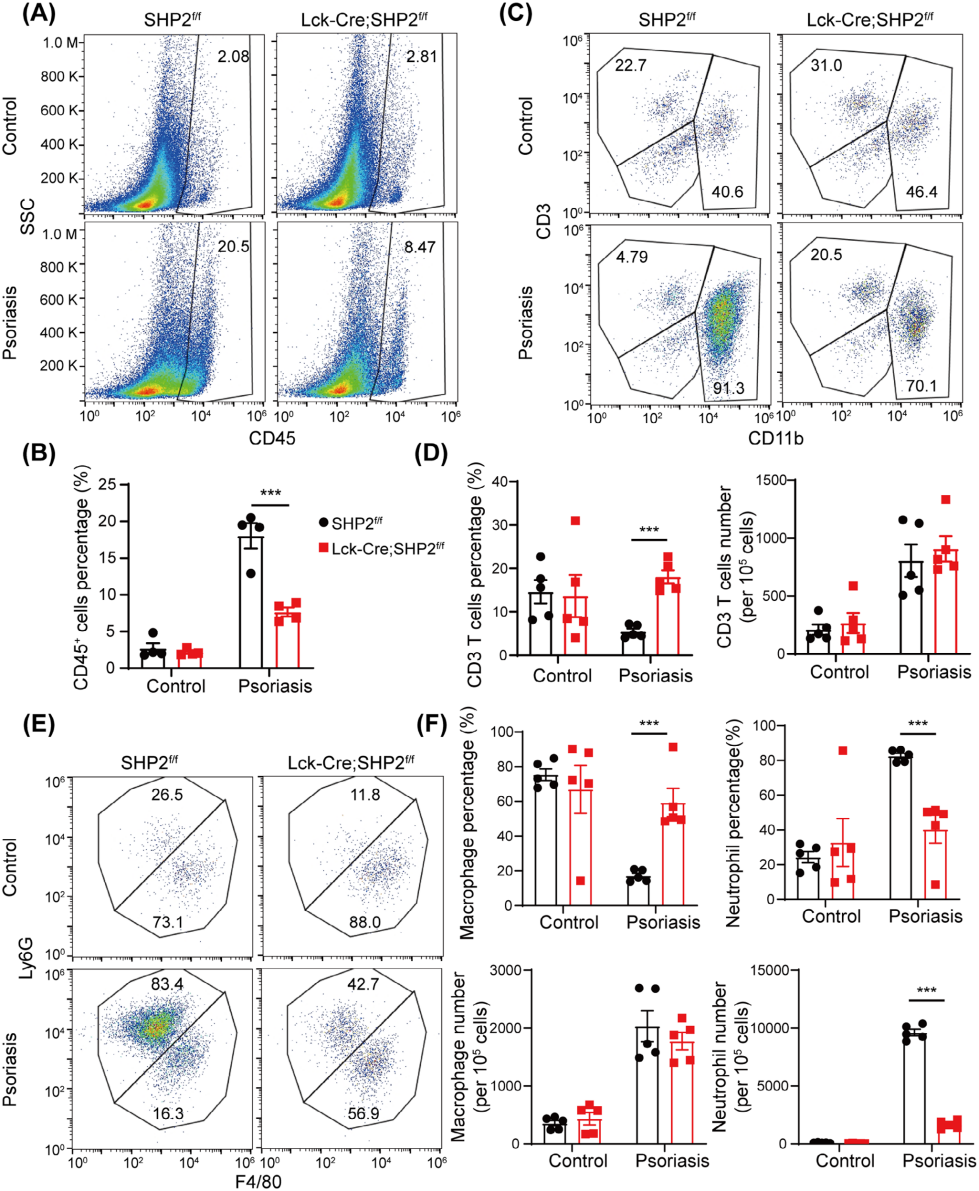
SHP2 deficiency in T cells regulates the immune profile of psoriasis‐like skin. (A) Flow cytometry analysis of CD45^+^ immune cells in skin tissue of mice. (B) Quantitative statistical chart of (A). *n* = 4. (C) Flow cytometry analysis of CD3^+^ T cells and CD11b^+^ myeloid immune cells in skin tissue of mice. (D) Quantitative statistical chart of (C). *n* = 5. (E) Flow cytometry analysis of F4/80^+^ macrophages and Ly6G^+^ neutrophils among CD11b^+^ myeloid cells in skin tissue of mice. (F) Quantitative statistical chart of E. n = 5. ***p < 0.001.

Based on the above findings, we further explored whether SHP2 deficiency modulates the immune profile in psoriasis skin. Single cells were isolated from the skin tissue, and the immune cell composition was analyzed via flow cytometry. The results showed that the expansion of CD45^+^ immune cells in the psoriasis‐like skin tissue was significantly suppressed in *Lck*‐Cre;SHP2^f/f^ mice (Figure 3A,B). Flow cytometric analysis revealed an increased frequency of CD3^+^ T cells in psoriasis mice while leaving their absolute numbers unchanged (Figure 3C,D). Further analysis of myeloid cells showed that psoriasis‐like lesions induced a marked increase in the number of macrophage and neutrophil in the mice. However, SHP2 deficiency reduced the proportion and absolute count of neutrophils, which consequently resulted in a relative increase in macrophage proportion (Figure 3E,F). These results indicate that SHP2 deficiency in T cells regulates the immune profile of psoriasis mice and significantly suppresses immune cell infiltration.

### 2.3 SHP2 Is Involved in Treg Differentiation In Vivo and In Vitro

Building on the previous findings, we observed that T cell‐specific SHP2 deficiency modulates the immune landscape in psoriasis mice. Similar to other autoimmune diseases, the development of psoriasis is also affected by Treg/Th17 balance [20, 22]. Therefore, we further investigated the impact of SHP2 on Treg and Th17 cells.

**FIGURE 4:**
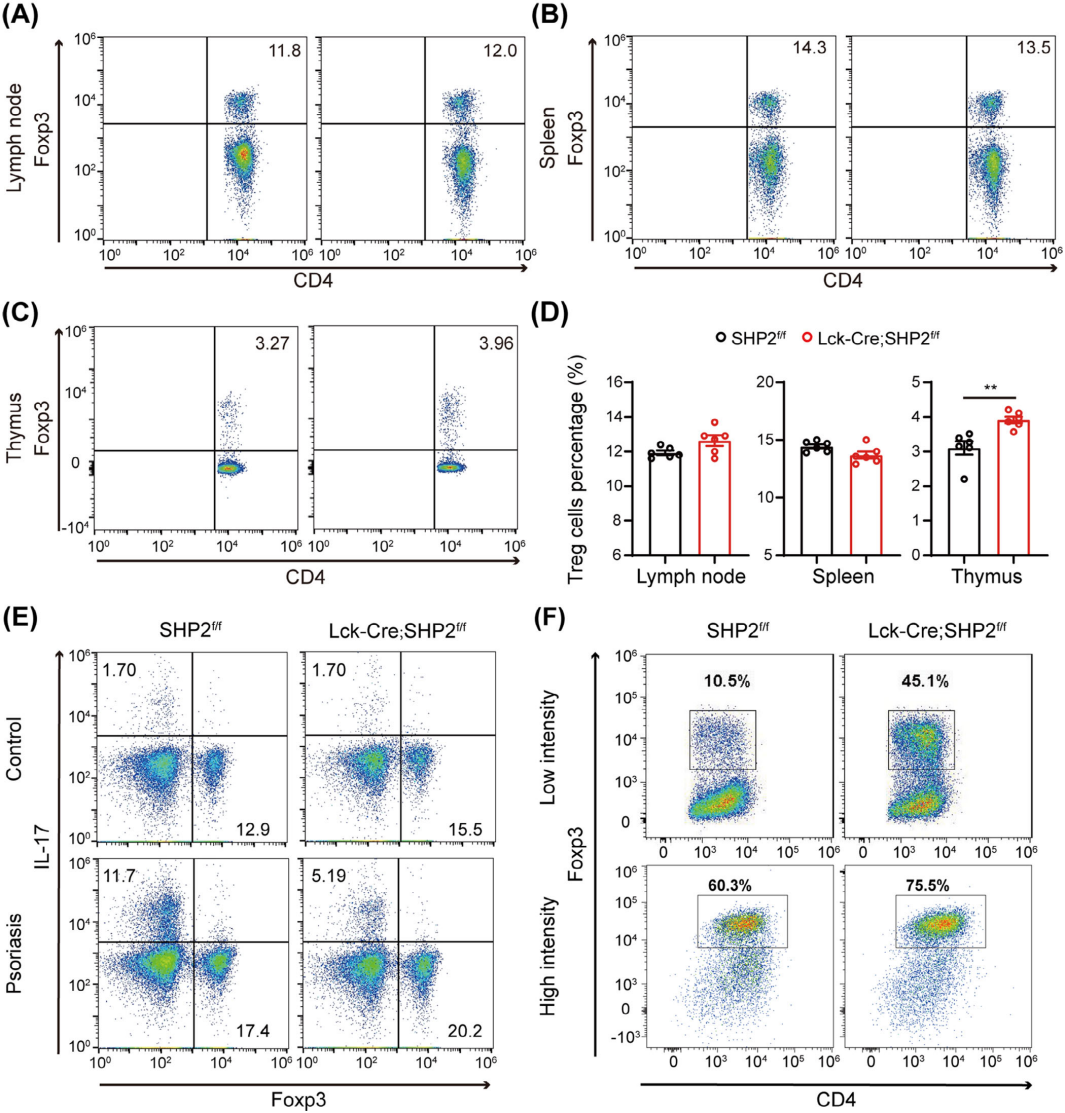
SHP2 is involved in Treg differentiation in vivo and in vitro. (A) Flow cytometry analysis of Tregs in the inguinal lymph nodes of normal mice. (B) Flow cytometry analysis of Tregs in the spleen of normal mice. (C) Flow cytometry analysis of Tregs in the thymus of normal mice. (D) Quantification of Treg percentages in inguinal lymph nodes, spleen, and thymus. *n* = 6. (E) Flow cytometry analysis of Treg and Th17 cells in skin‐draining lymph nodes in all groups. (F) Flow cytometry analysis of Treg differentiation induced from spleen‐derived naïve T cells of mouse under low‐intensity (5 ng/mL rIL‐2 and 4 ng/mL rTGF‐β) and high‐intensity stimulation (20 ng/mL rIL‐2 and 10 ng/mL rTGF‐β) in vitro.

First, we tested the level of baseline Treg in normal mice, and the results showed that all the proportion of Treg in lymph nodes, spleen, and thymus were comparable between SHP2^f/f^ and *Lck*‐Cre;SHP2^f/f^ mice under homeostatic conditions (Figure 4A–D), indicating no intrinsic defect in Treg development in the absence of inflammation. However, in IMQ‐induced psoriasis mice, SHP2 deficiency suppressed the expansion of Th17 cells while increasing the proportion of Tregs (Figure 4E). These results suggested that SHP2 deficiency selectively modulates Treg differentiation and Th17 cell accumulation specifically under pathological conditions.

To further validate the role of SHP2 in Treg differentiation, we conducted in vitro experiments using CD4^+^CD62L^+^ naïve T cells isolated from the spleens of SHP2^f/f^ and *Lck*‐Cre;SHP2^f/f^ mice. These cells were cultured under Treg‐inducing conditions, and differentiation potential was assessed. The results showed that *Lck*‐Cre;SHP2^f/f^ mice‐derived naïve T cells exhibited a stronger potential for differentiation into Tregs compared to SHP2^f/f^ mice under both low‐intensity and high‐intensity stimulation, with a marked increase in Treg frequency post‐induction (Figure 4F). The results show that SHP2 negatively regulates Treg differentiation both in vivo and in vitro.

### 2.4 SHP2 Deficiency Attenuates Colitis Induced by CD4^+^CD25^−^CD45RB^high^ T Cells

**FIGURE 5:**
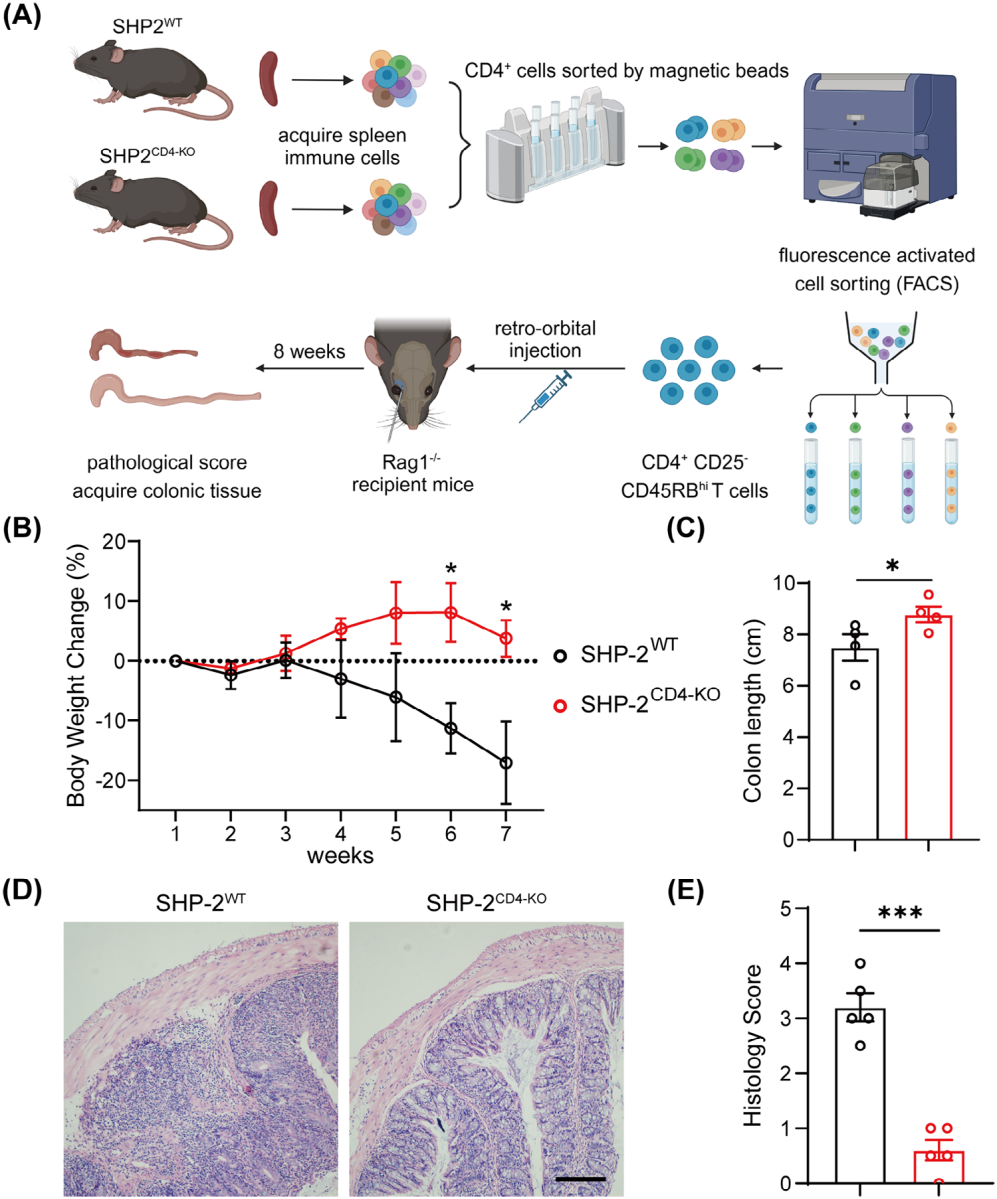
SHP2 deficiency attenuates colitis induced by CD4^+^CD25^−^CD45RB^high^ T cells. (A) Flow chart of modeling adoptive transfer colitis in mice. (B) Body weight change of recipient mice of CD4^+^CD25^−^CD45RB^high^ T cells from SHP2^WT^ and SHP2^CD4‐KO^ mice. (C) Colon length of recipient mice. *n* = 4. (D) HE staining of colon tissue of recipient mice, scale bar = 50 µm. (E) Histology scores of colonic tissues from recipient mice. *n* = 5. *p < 0.05, ***p < 0.001.

To confirm the regulatory effect of SHP2 on Treg differentiation, we established an adoptive transfer colitis model by transferring CD4^+^CD25^−^CD45RB^high^ T cells into Rag1^−/−^ recipient mice (Figure 5A). Compared to the Rag1^−/−^ mice receiving CD4^+^CD25^−^CD45RB^high^ T cells from SHP2^f/f^ donors, recipients of cells from *CD4*‐Cre;SHP2^f/f^ mice exhibited attenuated colitis severity, as evidenced by reduced weight loss (Figure 5B) and diminished colon shortening (Figure 5C). HE staining also showed that adoptive transfer of SHP2^f/f^ mice‐derived CD4^+^CD25^−^CD45RB^high^ T cells resulted in severe disruption of colonic villus architecture, intestinal wall thickening, and pronounced immune cell infiltration in the colon (Figure 5D). Generally, the recipient mice receiving CD4^+^CD25^−^CD45RB^high^ T cells from *CD4*‐Cre;SHP2^f/f^ mice displayed significantly lower histological scores (Figure 5E). These results suggest that the pathogenicity of CD4^+^CD25^−^CD45RB^high^ T cells is reduced by SHP2 deficiency.

### 2.5 SHP2 Restrains Treg Differentiation

**FIGURE 6:**
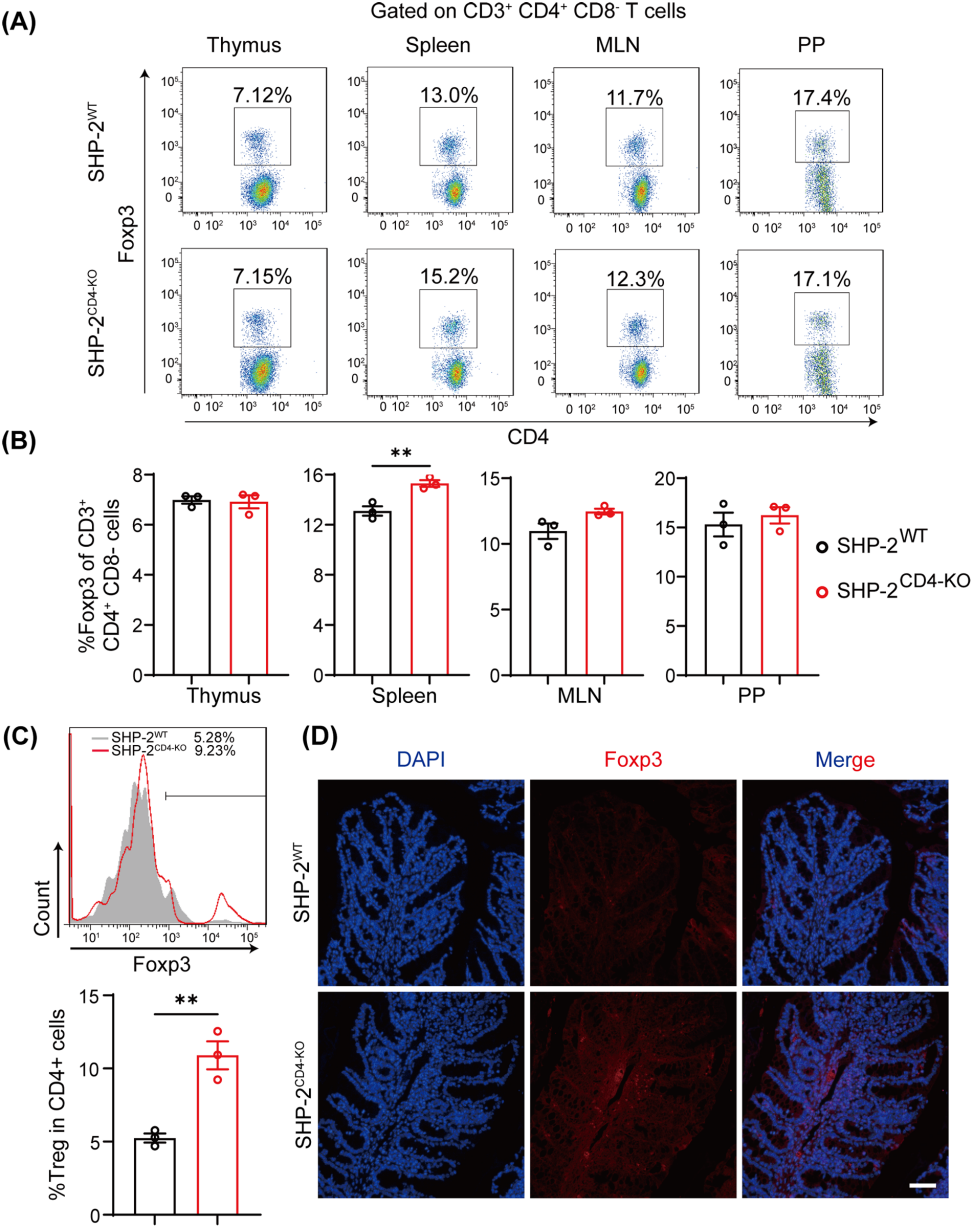
SHP2 restrains Treg differentiation. (A) Flow cytometry analysis of background Treg in thymus, spleen, mesenteric lymph nodes, and Peyer's patches of SHP2^WT^ and SHP2^CD4‐KO^ donor mice. (B) Quantitative statistical chart of (A). *n* = 3. (C) Flow cytometry analysis and quantification of Treg in mesenteric lymph nodes of recipient mice and fluorescence intensity of CCR6 expression on Treg surface. *n* = 3. (D) Immunofluorescence results of Foxp3^+^ Tregs in molding colon tissue, scale bar = 50 µm. **p < 0.01.

To determine whether the effect of SHP2 on CD4^+^CD25^−^CD45RB^high^ T cells’ pathogenicity is mediated by the regulation of Treg differentiation, we separately examined Treg levels in donor and recipient mice. We first investigated the effect of SHP2 deficiency on baseline Treg levels in donor mice. Flow cytometry revealed only marginal alterations in Treg proportions within mesenteric lymph nodes and Peyer's patches of *CD4*‐Cre;SHP2^f/f^ mice compared to SHP2^f/f^ controls (Figure 6A,B).

Flow cytometry analysis of T cells from adoptive transfer recipient mice revealed that the number and proportion of Tregs in lymph nodes of recipient mice receiving *CD4*‐Cre;SHP2^f/f^ mice‐derived CD4^+^CD25^−^CD45RB^high^ T cells were significantly elevated (Figure 6C). Similarly, a tangible increase in the abundance of Foxp3^+^ Tregs was also found in the colon tissue of recipient mice receiving *CD4*‐Cre;SHP2^f/f^ mice‐derived CD4^+^CD25^−^CD45RB^high^ T cells (Figure 6D). These data together suggest that SHP2 deficiency promotes pathogenic CD4^+^CD25^−^CD45RB^high^ T cell to differentiate into Tregs.

### 2.6 SHP2 Deficiency Enhances the Immunosuppressive Function of Treg

The results above demonstrated that SHP2 deficiency upregulated the proportion of Treg in adoptive transfer colitis mice, while whether the immunosuppressive function of these Tregs is altered remained unclear. To address this, we next evaluated the suppressive capacity of SHP2‐deficient Tregs in vivo and in vitro.

**FIGURE 7:**
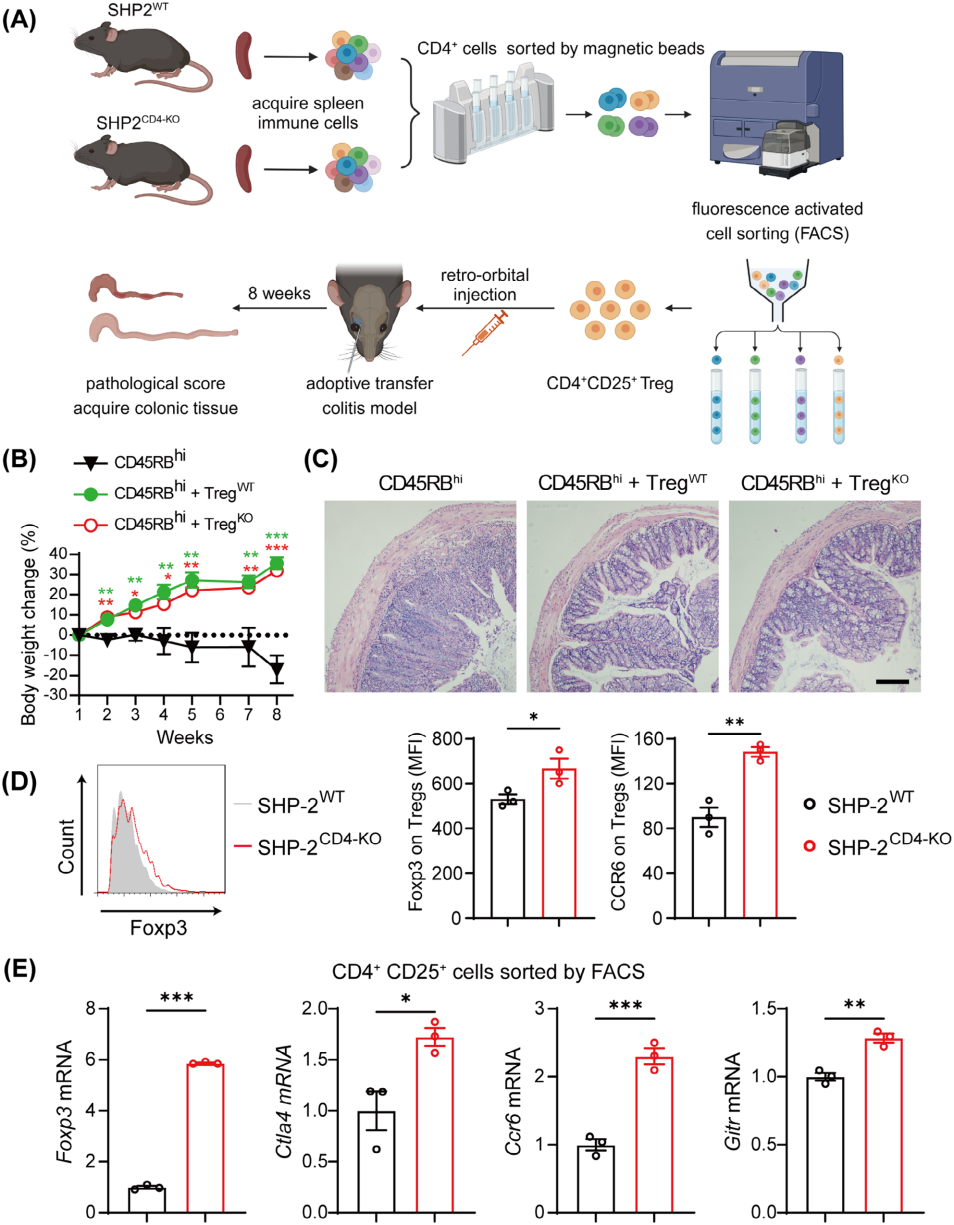
SHP2 deficiency enhances the immunosuppressive function of Treg. (A) Flow chart of modeling adoptive transfer colitis mice treated by receiving Treg from SHP2^WT^ and SHP2^CD4‐KO^ mice. (B) Weight change of adoptive transfer colitis mice after transferring Treg. (C) HE staining of colonic tissue of adoptive transfer colitis mice with Treg treatment, scale bar = 50 µm. (D) Flow cytometry analysis of Treg in spleen of recipient mice. *n* = 3. (E) Expression of *Foxp3*, *Ctla4*, *Gitr*, and *Ccr6* of CD4^+^CD25^+^ cells sorted by flow cytometry. *n* = 3. *p < 0.05, **p < 0.01, ***p < 0.001.

In vivo, we transferred equivalent numbers of CD4^+^CD25^+^ Tregs derived from SHP2^f/f^ and *CD4*‐Cre;SHP2^f/f^ mice into recipient mice that had received CD4^+^CD25^−^CD45RB^high^ T cells, respectively (Figure 7A). The results showed that CD4^+^CD25^+^ Treg from both groups equally suppressed disease progression in adoptive transfer colitis models, as evidenced by similarly reduced weight loss (Figure 7B) and ameliorated colonic lesions (Figure 7C). These results indicate that SHP2‐deficient Tregs retain immunosuppressive function. Further analysis of spleens from recipient mice via flow cytometric assessment demonstrated that SHP2 deficiency not only upregulated the number of Treg but also significantly increased the levels of Foxp3 and CCR6 on Tregs (Figure 7D). Additionally, the expressions of *Foxp3*, *Ctla4*, *Gitr*, and *Ccr6* genes in CD4^+^CD25^+^ Treg sorted by flow cytometry confirmed that the immunosuppressive capacity of Tregs was significantly enhanced upon SHP2 deficiency (Figure 7E). Together, these results demonstrate that SHP2 deficiency in T cells enhances the immunosuppressive function of Treg.

### 2.7 SHP2 Deficiency Intensifies STAT5/Smad3 Phosphorylation to Promote Treg Differentiation

**FIGURE 8:**
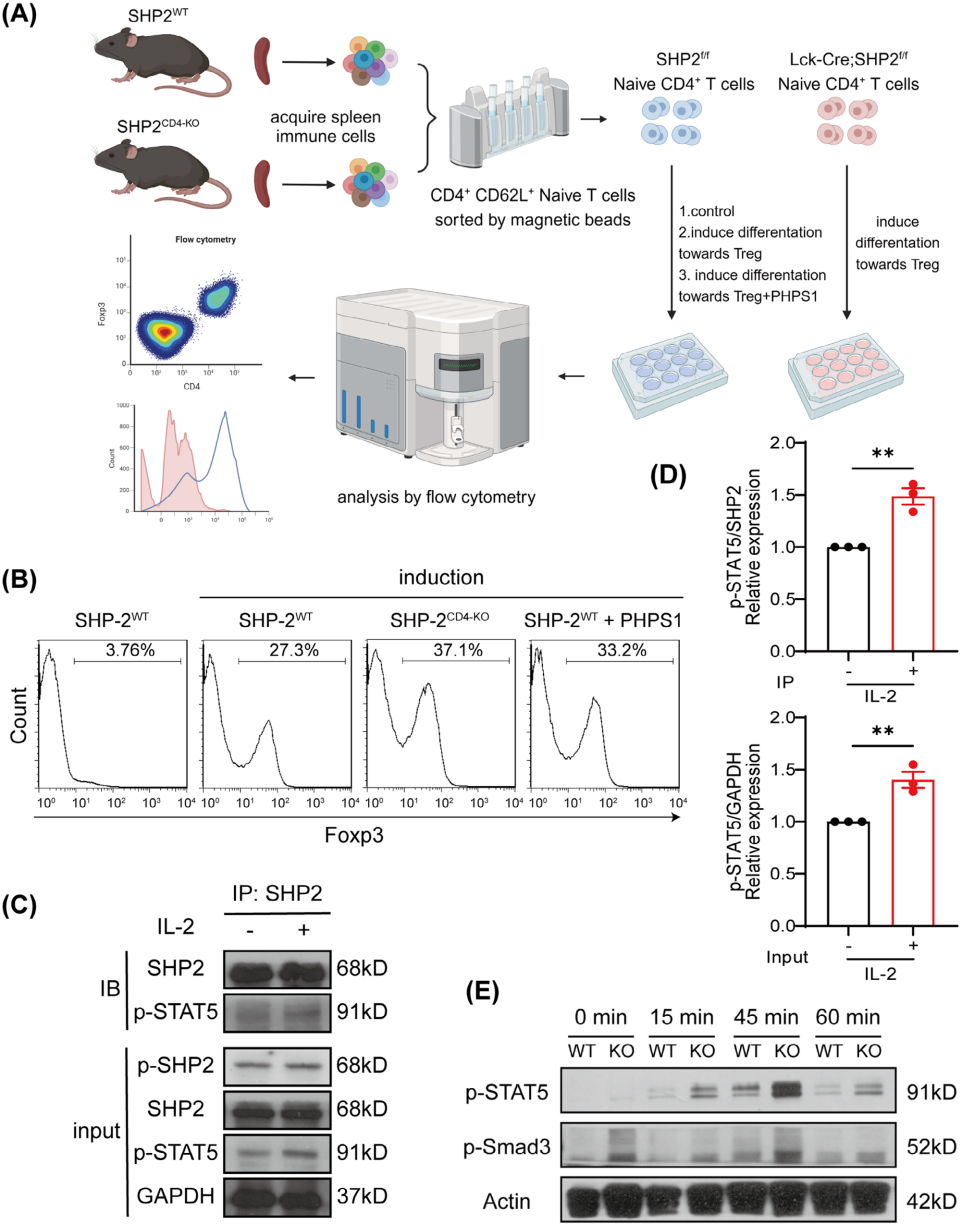
SHP2 deficiency intensifies STAT5/Smad3 phosphorylation to promote Treg differentiation. (A) Flow chart of splenic CD4^+^ naïve T cells obtained from SHP2^WT^ and SHP2^CD4‐KO^ donor mice and induced Treg differentiation in vitro. (B) Flow cytometry analysis of splenic CD4^+^ naïve T cells from SHP2^WT^ and SHP2^CD4‐KO^ donor mice after induction of Treg differentiation with or without PHPS1 (5 µM). (C) Immunoprecipitation between SHP2 and p‐STAT5. (D) Quantification of gray density in (C). (E) Phosphorylation of STAT5 and Smad3 in splenic CD4^+^ naïve T cells from SHP2^WT^ and SHP2^CD4‐KO^ donor mice after induction of Treg differentiation. **p < 0.01.

Having confirmed that SHP2 deficiency promotes Treg differentiation in vivo and in vitro, we used the phosphatase inhibitor of SHP2, PHPS1, to explore whether the effect of SHP2 on Treg differentiation depends on its phosphatase function. CD4^+^CD62L^+^ naïve T cells were isolated from the spleens of SHP2^f/f^ and *CD4*‐Cre;SHP2^f/f^ mice, respectively, and were further induced to differentiate toward Treg in vitro in the presence or absence of PHPS1 (Figure 8A). Flow cytometric analysis showed that either knockout of SHP2 or PHPS1‐mediated SHP2 inhibition significantly promoted the differentiation of naïve T cells into Tregs compared to untreated controls (Figure 8B).

Previous studies have shown that Smad and STAT5 signaling, downstream of TGF‐β and IL‐2, are critical for Treg differentiation and the Th17/Treg balance [40], and the phosphorylation of SMAD3 and STAT5 in T cells induced FOXP3 transcription and mediated Treg amplification [41]. Furthermore, SHP2 can directly dephosphorylate STAT5 in vitro [42]. Immunoprecipitation results showed that SHP2 could bind to STAT5 and participate in its phosphorylation process (Figure 8C,D). To further explore this mechanism, we examined the phosphorylation levels of STAT5 and Smad3 in spleen‐derived CD4^+^CD62L^+^ naïve T cells from SHP2^f/f^ and *CD4*‐Cre;SHP2^f/f^ mice following Treg differentiation induction. The results showed that SHP2 deficiency indeed significantly upregulates phosphorylation of STAT5/Smad3 (Figure 8E). These results suggest that SHP2 deficiency enhances Treg differentiation by promoting STAT5/Smad3 phosphorylation.

## 3 Discussion

Psoriasis is a common, recurrent, chronic skin disease characterized by skin inflammation caused by immune system dysfunction. As with many autoimmune conditions, the dysregulation of Th17/Treg equilibrium is a central pathogenic mechanism in psoriasis [24, 25, 43, 44]. Despite prior studies on SHP2's role in autoimmune diseases, its influence on Th17/Treg homeostasis remained poorly defined. Building on these observations, we hypothesized that SHP2 might modulate autoimmune disease progression by directly regulating Treg differentiation and expansion.

Initially, we found that the IMQ‐induced *Lck*‐Cre;SHP2^f/f^ mice exhibited a milder psoriasis‐like phenotype compared to SHP2^f/f^ mice, suggesting that SHP2 in T cell may contribute to the pathogenesis of psoriasis (Figures 1 and 2). Subsequent studies revealed that SHP2‐specific deletion regulates the immune profile of psoriasis mice, specifically reducing infiltration of immune cells, upregulating the proportion of Treg while downregulating the proportion of Th17 cells in psoriasis mice (Figures 3 and 4). These results indicated that inhibiting SHP2 could alleviate psoriasis through regulating the balance of Th17/Treg. In fact, the positive effects of SHP2 inhibitors on the treatment of psoriasis have also been illustrated in our previous studies [38, 45, 46]. For instance, SHP2 was shown to drive neutrophil extracellular trap (NET) formation via the ERK5 pathway, which enhances pro‐inflammatory cytokine release, amplifies neutrophil–keratinocyte crosstalk, and exacerbates psoriasis‐like skin lesions [38]. Furthermore, SHP2 promotes the ubiquitination of toll‐like receptor 7 (TLR7) and NF‐κB‐mediated psoriasis‐like skin inflammation by dephosphorylating TLR7 in macrophages. While the allosteric inhibitor of SHP2, SHP099, or myeloid‐specific SHP2 knockout could both improve IMQ‐induced psoriasis‐like skin inflammation in mice [45]. Additionally, another allosteric inhibitor of SHP2, TK‐453, could also alleviate psoriasis‐like skin inflammation by suppressing the IL‐23/Th17 axis [46]. These studies together highlight that SHP2 is broadly expressed in various immune cells and exerts multifaceted roles in inflammation, while targeted inhibition of SHP2 can ameliorate psoriasis from multiple mechanisms, including regulating Treg differentiation.

To further confirm the regulatory role of SHP2 in Treg differentiation, we established an adoptive transfer colitis model by transferring CD4^+^CD25^−^CD45RB^high^ T cells from SHP2^f/f^ and *CD4*‐Cre;SHP2^f/f^ mice to Rag1^−/−^ mice, thereby isolating the influence of CD4+ T cells on disease onset and progression. The results revealed that SHP2‐deficient CD4^+^CD25^−^CD45RB^high^ T cells exhibited weakened pathogenicity, manifested as alleviated colitis symptoms in recipient mice (Figure 5). Subsequently, we separately assessed the Treg proportion in donor and recipient mice. Under the healthy state, the Treg of donor mice was almost not affected by the deletion of SHP2. However, in the colitis recipient mice that received the naïve T cells from *CD4*‐Cre;SHP2^f/f^ mice, the proportion of Treg significantly increased. These findings suggest that SHP2 exerts minimal influence on Treg differentiation under physiological conditions but strongly promotes Treg expansion in pathological contexts (Figure 6). In the T cell transfer colitis model, transferred Tregs are recruited to the mesenteric lymph nodes, submucosal layer, solitary lymphoid follicles, and spleen in the colon. Continuous immune responses activate Tregs, thereby suppressing both systemic and local inflammatory activity, accompanied by a decreased number of pathogenic T cells and effector innate immune cells [47]. Therefore, in order to further investigate the effect of SHP2 on the immunosuppressive function of Treg, we respectively transferred Treg from SHP2^f/f^ and *CD4*‐Cre;SHP2^f/f^ mice into model mice. As expected, SHP2‐deficient Tregs displayed significantly enhanced suppressive activity (Figure 7). In vitro experiments further demonstrated that SHP2 may play a role through its phosphatase function. Concretely, SHP2‐specific deletion promotes Treg differentiation by enhancing STAT5/Smad3 phosphorylation (Figure 8).

Many anti‐inflammatory cytokines are central to maintaining immune homeostasis and healing, which are also common immune triggers for inflammatory bowel disease and psoriasis. The core driver of psoriatic inflammation is the IL‐23/Th17 axis. In the immunological etiology of inflammatory bowel disease, the translocation of gut microbiota across the intestinal barrier may primarily stimulate ongoing T cell responses via antigen‐presenting cells in the gut [8]. Despite overlapping therapeutic approaches such as immunosuppressive drugs, psoriasis and colitis exhibit significant heterogeneity in treatment efficacy. In psoriasis, therapeutic strategies targeting IL‐23 and IL‐17 have shown superior efficacy over TNF inhibitors, while the former may be less beneficial in inflammatory bowel disease [48, 49, 50]. One plausible explanation could be that Th17 has a significant physiological function in the gut and appears to support barrier integrity and immunological balance through IL‐22 production, increased antimicrobial peptide release, and tight junction expression [51, 52]. Given the complexity of Th17 in psoriasis and colitis, we prioritized studying Treg that is universal and stable in autoimmune diseases rather than Th17 in this study.

Abnormal Tregs lead to the occurrence of various autoimmune diseases such as psoriasis, inflammatory bowel disease, multiple sclerosis, systemic lupus erythematosus, and rheumatoid arthritis [20, 53, 54], while targeting Tregs to influence physiological and pathological immune responses, such as depleting them to boost tumor immunity or engineering and expanding them to treat immunological disorders, could be feasible [55, 56]. Inevitably, there are also some limitations in this study. First, only psoriasis and colitis, but no other animal models, such as systemic lupus erythematosus, rheumatoid arthritis, and multiple sclerosis, were involved in this research. Therefore, more direct evidence is lacking to extend the conclusion to all autoimmune diseases. Second, due to the complex function of Th17 in colitis, this study rarely explored the changes of Th17 but only focused on the effect of SHP2 on Tregs.

In this study, our results showed that in both psoriasis and colitis, the deletion of SHP2 in T cells could promote the proliferation of Treg. This phenomenon is very likely to be common in autoimmune diseases, suggesting that SHP2 may become a promising common therapeutic target in the treatment of autoimmune diseases by interfering with the differentiation of Treg.

## 4 Materials and Methods

### 4.1 Mice

*Lck*‐Cre;SHP2^f/f^ (*Lck*‐Cre;*Ptpn11* ^f/f^) and SHP2^CD4‐KO^ (*CD4*‐Cre;SHP2^f/f^) mice were generated by crossing *Ptpn11* ^f/f^ mice with mice expressing Cre driven by endogenous *Lck* and *CD4* promoter, respectively. All mice were sourced from GemPharmatech Co. Ltd. (Nanjing, China) and maintained on a C57BL/6 background. Animals were housed under specific pathogen‐free conditions at 21°C with a 12‐h light–dark cycle, without food or water restrictions. The *Guide for the Care and Use of Laboratory Animals* (National Institutes of Health, USA) and our university's associated ethical guidelines were followed when it came to animal care and experimental procedures.

#### 4.1.1 IMQ‐Induced Psoriasis Mice

SHP2^f/f^ and *Lck*‐Cre;SHP2^f/f^ mice were randomly divided into two groups labeled as control and IMQ‐induced psoriasis. The 2 cm × 2 cm of skin on the back of mice was shaved and exposed to Vaseline cream (control) and IMQ continuously for 5 days.

#### 4.1.2 Adoptive Transfer Colitis Mice and Treg Treatment

All splenic cells of SHP2^WT^ and SHP2^CD4‐KO^ mice were obtained under sterile conditions, respectively. CD4^+^CD25^−^CD45RB^high^ T cells and CD4^+^CD25^+^ Treg were sorted by magnetic CD4 MicroBeads (Miltenyi Biotec, 130‐117‐043) followed by fluorescence‐activated cell sorting (FACS) through PE‐anti‐CD4, FITC‐anti‐CD45RB, and APC‐anti‐CD25.

After exposure to isoflurane inhalant anesthesia, Rag1^−/−^ recipient mice were transferred with 100 µL of CD4^+^CD25^−^CD45RB^high^ T cells (2.5 × 10^6^ cells per mL) from SHP2^WT^ and SHP2^CD4‐KO^ mice through retro‐orbital injection, respectively, to induce an adoptive transfer colitis model.

For Treg treatment, recipient mice were randomly divided into three groups. Together with CD4^+^CD25^−^CD45RB^high^ T cells isolated from the spleen of C57BL/6 mice, 100 µL cell suspension containing 4 × 10^4^ Tregs from SHP2^WT^ and SHP2^CD4‐KO^ mice was, respectively, transferred into Rag1^−/−^ recipient mice at the same time.

### 4.2 Cells

CD4^+^CD62L^+^ T Cell Isolation Kit (Miltenyi Biotec, 130‐106‐643) was used to isolate CD4^+^CD62L^+^ naïve T cells from the spleen of mice. CD4^+^CD62L^+^ naïve T cells were seeded into 96‐well plates with a density of 2 × 10^4^ cells per well to differentiate toward Treg in vitro. The cells were cultivated in RPMI1640 medium containing anti‐CD3e (2 µg/mL), anti‐CD28 (0.5 µg/mL), 2‐mercaptoethanol (55 µM, Invitrogen), and 10% fetal bovine serum. For iTreg, recombinant IL‐2 (5 or 20 ng/mL under low/high‐intensity stimulation), recombinant TGF‐β (4 or 10 ng/mL under low/high‐intensity stimulation), and anti‐IFN‐γ (1 µg/mL) antibodies were added. FACS test samples 5 days later.

### 4.3 Pathological Scoring

The severity of the psoriasis model was measured by the pathological score according to the erythema and scaling, which was scored from 0 to 4: 0, none; 1, slight; 2, moderate; 3, marked; and 4, very marked. The severity of the adoptive transfer model was measured by the histology score according to diarrhea and hematochezia, which was scored from 0 to 4: 0, none; 1, slight; 2, moderate; 3, marked; and 4, very marked.

### 4.4 Quantitative PCR (qPCR)

Following the manufacturer's instructions, TRIzol (TaKaRa) was used to extract total RNA, which was further converted into single‐stranded cDNA through reverse transcription. The program for PCR amplification was as follows: 94°C for 3 min, 35 cycles of 94°C for 30 s, 62°C for 40 s, and 72°C for 1 min. The data were normalized using β‐actin expression. Primers used are shown in Table S1.

### 4.5 Western Blotting

Cell lysis buffer for Western and IP (Beyotime, P0013) including protease and phosphatase inhibitor cocktails (Roche, PhosSTOP) was used to extract the proteins. SDS‐polyacrylamide gel electrophoresis was used to separate the proteins measured by the BCA assay, which were then electrophoretically transferred onto polyvinylidene difluoride membranes. After overnight antibody probing at 4°C, the membranes were incubated with a secondary antibody linked to horseradish peroxidase. Protein expression was detected using x‐ray film developing equipment. Antibodies used are shown in Table S2.

### 4.6 Immunofluorescence Staining

After being dewaxed and hydrated, slices embedded in paraffin were heated for 2 min in citrate antigen retrieval solution (Beyotime, P0081). The slices were blocked with 5% goat serum and then respectively treated with antibodies at 4°C for the entire night and the secondary antibody at room temperature for 1 h. Finally, slices were stained with DAPI solution. Antibodies used are shown in Table S2.

### 4.7 Immunoprecipitation

Single cells from murine spleen and inguinal lymph nodes were harvested and equally divided into two culture dishes, which were pre‐coated with anti‐CD3 antibody (2 µg/mL) and anti‐CD28 antibody (0.5 µg/mL). Cells were cultured with RPMI 1640 medium containing 10% FBS, which was changed every 2 days. After 4 days, one dish was added with 5 ng/mL recombinant mouse IL‐2 (PeproTech), while another was not. Cells were harvested after 30 min to obtain protein for Western blotting.

### 4.8 Isolation of Single Cells From Murine Tissues

Mice were sacrificed, and the dorsal skin was cut into fragments, washed three times with cold PBS, and transferred to RPMI 1640 medium containing 2 mg/mL Collagenase I (Sigma, 1148089), 2 mg/mL Collagenase II (Sigma, 1148090), 3 mg/mL Dispase II (Sigma, D4693), and 10 µg/mL DNase I (Sigma, 11284932001) for digestion at 37°C in a shaker for 90 min. All the liquid and tissue were strained through a 70‐µm mesh into the 15‐mL tube, and 5 mL RPMI 1640 medium was added to rinse the cells. Single‐cell pellets of skin were harvested after centrifugation at 4°C, 300 g, for 5 min and resuspended in RPMI 1640 medium.

The peritoneum of the mice was opened, exposing the abdominal cavity and thorax, and the spleen, thymus, Peyer's patches, and mesenteric lymph nodes were exhibited. Tissues were put in cold, sterile PBS and thoroughly ground with the handle of a sterile syringe. The tissue solution mixture was strained through a 70‐µm mesh to obtain a complete cell suspension. Erythrocytes were lysed in the spleen with Tris‐NH_4_Cl, which was not necessary for inguinal lymph nodes, the thymus, Peyer's patches, and mesenteric lymph nodes.

### 4.9 Flow Cytometry

For single‐cell surface staining, cells were incubated with fluorescently labeled antibodies on ice in the dark for 30 min, followed by washing and detection. For intracellular staining, cells were cultured in RPMI 1640 medium supplemented with 10% FBS and Cell Stimulation Cocktail (eBioscience, 00‐4975‐93) for 4 h before surface staining. Subsequently, cells were fixed and permeabilized according to the instructions of the FOXP3 Fixation/Permeabilization Kit (eBioscience, 00‐5523‐00) and then stained for the intracellular cytokines FOXP3 and IL‐17A. A FACSaria II (BD) or Attune NxT (Life Technologies) flow cytometer was used to collect the data, and FACSDiva or FlowJo (BD) software was used for analysis. Antibodies used are shown in Table S3.

### 4.10 Statistics

Statistical analyses were conducted using GraphPad Prism 8.3.0. The statistical significance between the two groups was determined using a two‐tailed Student's *t*‐test. A one‐way ANOVA test was used to assess significant differences among the three groups. Flow cytometry results were analyzed using Flowjo_V10. *p* < 0.05 was considered to be statistically significant. *p < 0.05, **p < 0.01, ***p < 0.001.

## Author Contributions

X.W., Y.S., and F.S. conceived and supervised the study. S.Z., Z.O., and Z.F. performed experiments and analyzed the data. H.S. and H.Y. provided the experimental materials. S.Z. and F.S. wrote the manuscript. All authors discussed the results and commented on the manuscript.

## Ethics Statement

Animal care and experimental procedures were carried out in compliance with the Institutional Animal Care and Use Committee of Nanjing University (Approval number #IACUC‐230036).

## Conflicts of Interest

The authors declare no conflicts of interest.

## Supporting information




**Supplementary File 1**: Table S1. Primers used for RT‐qPCR.
Table S2. Antibodies used for immunofluorescence and western blot.
Table S3. Antibodies used for flow cytometry.

## Data Availability

Data will be made available on request.
