# Supplementary material for: T Cell‐Specific Deficiency of Src Homology 2‐Containing Protein Tyrosine Phosphatase 2 Ameliorates Psoriasis and Colitis by Promoting Treg Differentiation
Source: MedComm (2020). 2025 Aug 1;6(8):e70310. doi: 10.1002/mco2.70310 (PMC12314549; doi:10.1002/mco2.70310)
Supplement: Supplementary file 1 — Table S1. Primers used for RT‐qPCR. Table S2. Antibodies used for immunofluorescence and western blot. Table S3. Antibodies used for flow cytometry. [file MCO2-6-e70310-s001.docx]

**T cell-specific deficiency of** **Src homology 2-containing protein tyrosine phosphatase 2 ameliorates psoriasis and colitis by promoting Treg differentiation**

Shuqiong Zhang^1,2,#^, Zijun Ouyang ^3,#^, Zhidan Fan^4,#^, Haiyan Sun^3^,

Haiguo Yu^4^, Xingxin Wu^1,^*, Yang Sun^1,2,^*, Fenli Shao^5,^*.

^1^ State Key Laboratory of Pharmaceutical Biotechnology, Chemistry and Biomedicine Innovation Center (ChemBIC), School of Life Sciences, Nanjing University, 163 Xianlin Avenue, Nanjing 210023, Jiangsu, China;

^2^ Jiangsu Key Laboratory of New Drug Research and Clinical Pharmacy, Xuzhou Medical University, 209 Tongshan Road, Xuzhou 221004, China;

^3^ School of Food and Drug, Shenzhen Polytechnic University, 7098 Liuxian Avenue, Shenzhen 518055, China;

^4^ Department of Rheumatology and Immunology, Children’s Hospital of Nanjing Medical University, 72 Guangzhou Road, Nanjing 210008, China;

^5^ State Key Laboratory of Technologies for Chinese Medicine Pharmaceutical Process Control and Intelligent Manufacture，Nanjing University of Chinese Medicine，Nanjing 210023, China.

* Corresponding authors.

E-mail addresses: [yangsun@nju.edu.cn](mailto:yangsun@nju.edu.cn); xingxin.wu@nju.edu.cn; [fenlishao@njucm.edu.cn](mailto:fenlishao@njucm.edu.cn).

^#^ These authors contributed equally to this work.

E-mail addresses: [ZSQ_SHERRY@163.com](mailto:ZSQ_SHERRY@163.com); [ouyangzijun@szpu.edu.cn](mailto:ouyangzijun@szpu.edu.cn).

[zhidan1728@163.com](mailto:zhidan1728@163.com)

**This file includes:**

**Table S1 to S3**

**Table S1. Primers used for RT-qPCR.**

| **Gene** | **Forward-sequence** | **Reverse-sequence** |
| --- | --- | --- |
| Actin | GGCTGTATTCCCCTCCATCG | CCAGTTGGTAACAATGCCATGT |
| Tnfα | CCCTCACACTCAGATCATCTTCT | GCTACGACGTGGGCTACAG |
| Il17a | TTTAACTCCCTTGGCGCAAAA | CTTTCCCTCCGCATTGACAC |
| S100a7 | TGCTCTTGGATAGTGTGCCTC | GCTCTGTGATGTAGTATGGCTG |
| Krt16 | GGTGGCCTCTAACAGTGATCT | TGCATACAGTATCTGCCTTTGG |
| Foxp3 | CCCATCCCCAGGAGTCTTG | ACCATGACTAGGGGCACTGTA |
| Ctla4 | GCTTCCTAGATTACCCCTTCTGC | CGGGCATGGTTCTGGATCA |
| Gitr | GCCATGCTGTATGGAGTCTCG | CCACTTCCGTTCTGAACCTTG |
| Ccr6 | CCTGGGCAACATTATGGTGGT | CAGAACGGTAGGGTGAGGACA |

**Table S2. Antibodies used for immunofluorescence and western blot.**

| **Antibodies** | **Brand** | **ID** |
| --- | --- | --- |
| Phospho-Stat5 (Tyr694) (D47E7) Rabbit mAb | Cell Signaling Technology | 4322T |
| Phospho-SMAD3 (Ser423/425) (C25A9) Rabbit mAb | Cell Signaling Technology | 9520T |
| Mouse anti-β-actin | Abmart | M20011 |
| Mouse anti-GAPDH | Abmart | M20006M |
| Anti-Ki67 | Cell Signaling Technology | 9129T |
| goat anti-rabbit IgG conjugated to Alexa Fluor 488 | Invitrogen | A-11008 |

**Table S3. Antibodies used for flow cytometry.**

| **Antibodies** | **ID** | **Brand** |
| --- | --- | --- |
| Anti-Mouse CD16/CD32 | 14-0161-82 | eBioscience |
| PerCP-Cy5.5-anti-CD45 | 103131 | Biolegend |
| APC-Cy7-anti-CD3 | 100221 | Biolegend |
| PE-Cy7-anti-CD11b | 25-0112-82 | eBioscience |
| APC-anti-Ly6G | 17-9668-82 | eBioscience |
| PE-anti-F4/80 | 12-4801-82 | eBioscience |
| PE-Cy7-anti-CD4 | 116015 | Biolegend |
| FITC-anti-CD8a | 100705 | Biolegend |
| APC-anti-CD4 | 17-0042-82 | eBioscience |
| FITC-anti-CD25 | 102005 | Biolegend |
| PE-anti-Foxp3 | 12-5773-82 | eBioscience |
| APC-anti-IL-17A | 17-7177-81 | eBioscience |
| APC-anti-CD25 | 17-0257-42 | eBioscience |
| PE-anti-CD4 | 12-0041-82 | eBioscience |
| FITC-anti-CD45RB | 11-0455-82 | eBioscience |
| PE-Cy7-anti-Foxp3 | 25-5773-82 | eBioscience |
| BV605-anti-CD196(CCR6) | 129819 | Biolegend |
